# Insane Hospital Supervision

**Published:** 1886-04

**Authors:** E. C. Spitzka

**Affiliations:** New York; Late Professor of Anatomy of the Nervous System, New York Post-Graduate School of Medicine


THE CHICAGO

MEDICAL JOURNAL AND EXAMINER.

VOL. LIII. APRIL, 1886. No. 4.

### ORIGINAL COMMUNICATIONS.

### INSANE HOSPITAL SUPERVISION.

*Quis Custodit Custodes.*

[Read before the New York Medical Jurisprudence Society, February 11th, 1886.]

*By* E. C. SPITZKA, M. D., New York. *Late Professor of Anatomy of the Nervous System, New York Post-Graduate School of Medicine.*

It is not a great many years since a distinguished British alienist, commenting on the management of American insane hospitals, took occasion, with special reference to the Blockley alms-house insane-hospital, Philadelphia, and the kindred institutions on Blackwell's and Ward's Island, New York, to say that no American could contemplate their condition without a feeling of humiliation. He found that while the state hospitals bore comparison with the better class of similar institutions in Europe, the management of the county hospitals of the larger cities was so inseparably tied up with local politics as to be beneath criticism. On one occasion he had pointed out to him the President of the Charity Commission of one of our large cities; "his colleagues engaged in business, leaving almost all the power in his hands. He was a *politician*, in the American sense of the word, which is not complimentary, and had begun his official career as night-watch in a hospital," and this remarkable fact is cited as an apt illustration of the correctness of his inference. To us, who have seen the ex-night watchman riding in grand regalia at the head of the political processions with which we are occasionally afflicted, there is nothing very startling in it. On civil service grounds, perhaps a man who is capable of marshalling such a rank and file as comprise these processions, and who has served in the ranks himself, is just as fit to occupy an office which unsophisticated people are apt to consider only gentlemen and scholars eligible to—as a militia brigadier is fit to be President of the Board of Health. Still, in his main inference the distinguished foreign writer is correct; offices connected with the management of sanitary and humanitarian interests are too apt to be sought for by those who have failed in other departments of business or professional activity; they are often persons who seek through the devious channels of patronage what they could not obtain through merit and in healthy competition. The consequence is, the preponderance of non-expertness in the management of many of our institutions. The inmates are comparatively fortunate if immorality, corruption and positive brutality are not also engendered by the same system. Without making too specific and personal references, it may be said that even the medical offices of insane hospitals are not as free from these influences as they should be. The reason is, that men of character—and the true physician must be a man of the very highest character—do not usually possess the political requirements for political positions. When an ex-horse-car conductor and an ex-supervisor of a bathing establishment graduate into superintendents of insane-hospitals situated within a radius of fifteen miles of one of our large cities, it must be admitted that the percentage of those in office who have not had the best opportunities for acquiring an elaborate training in one of the most profound medical specialities is by no means small. Of the frivolous political reasons for making changes in insane-hospital medical care which have afflicted the United States, one of the most glaring is the following: The people of Virginia, in 1882, were divided on a state debt issue. Among those who favored the payment of the debt were all the superintendents of asylums in the state, and they were summarily dismissed from office on the success of the repudiation measure. Changes of this kind, which are exceedingly frequent in many states, naturally lead to the inquiry as to what means of supervision exist to detect the evils resulting from such political changes.

In every community there are a number of ladies and gentlemen who, besides leisure and wealth, possess a kind heart and a disposition to improve the condition of their less fortunate fellow beings. The prominence of these in the community and the influence they exert, may be almost claimed as peculiar to the Anglo-Saxon race. Howard, the English prison reformer, our own Howe, the educator of the deaf and dumb and philhellenist, the Earl of Shaftsbury, who became the English champion of the insane, as Miss Dix became the American, are examples of this. Efforts, sometimes spasmodic, sometimes enduring, are made by such philanthropists to neutralize the bad features attendant on the political managements of alms-houses, asylums and jails. Their inquiring spirit and reformatory tendencies soon earn them the stigma of being "cranks;" the politician views them with distrust if not with positive hatred; for the brutal prison task-master, the thieving alms-house supervisor, the negligent asylum attendant, are never quite secure against an unexpected visit and ensuing denunciation by some one of these people. It occurred to the Dædalian mind of some brilliant legislator to turn the stream of threatened investigation and reform into official channels, and "Boards of Charities" were organized. Thereafter, when any meddlesome individual undertook to criticise the food, the medical or other treatment of paupers, prisoners or the insane, he was told that he was misinformed; that abuses were impossible as long as there existed a State Board of Charities, composed of gentlemen of the very highest character. On many occasions, cruel or indolent or incompetent officials were able to shield themselves behind these "gentlemen of the very highest character." It has been well said by the late Dr. Wilbur: "The model on which our State Boards of Charities have been formed is that of the English Commissioners in Lunacy. To its high intelligence and ability, together with its entire independence of party and class interests, the acknowledged superiority of the English system of provision for the insane is chiefly due. . . . Yet the legal powers of these commissioners are as limited as those of our State Boards of Charities. They can do little but advise and remonstrate against abuses personally and through their reports. How is it the English Commissioners in Lunacy accomplish so much and our State Boards of Charities accomplish so little?" It must be admitted that, as a rule, State Boards of Charities are of unexceptionable composition, their members good men, and in the majority men of substance; still the difficulty lies in the organization of these bodies, the position of member on this board, as in England, is an unsalaried one, and there is but the technical obligation on him of performing any but the most perfunctory duties. There is one salaried officer, usually called a secretary, who in some cases owes his position to a long course of toadying to some political matadore, finally becomes the impersonation of the Board, and impresses on it all the objectionable characters of a political organization. It must be evident that under such conditions the State Boards of Charities too frequently serve as bulwarks to the politician, and in place of exposing abuses simply serve to strew dust in the eyes of the public by superficial inspections.

The inadequacy of these State Boards to perform these assigned functions is illustrated every year, and in almost every state. The Tewkesbury alms-house horrors developed under the fostering eyes of such a Board; the rowdy management of some county asylums in New York and New Jersey still continues, although it must in justice be admitted that in New York, Pennsylvania and Wisconsin whatever reform has been accomplished is due to the efforts of State Boards of Charities, fought as they were by an unscrupulous insane-hospital ring. Long ago recognizing the inadequacy of even the best State Boards of Charities to perform the duties incumbent on them, I suggested in 1878 that every large state should have three State Commissioners in Lunacy to whom the supervision of the insane should be relegated. I again suggest this, and with the view of illustrating the valueless character of the inspection of the average State Board, I have ventured to bring before you one glaring instance. In order to avoid the imputation of being personal in my references, I have selected one which exercises its functions a thousand miles distant from New York. I shall ask you to listen to the evidence, derived from and under the very auspices of this State Board itself; and if your verdict will not be that this body stands self-convicted of a violation of the "Golden Rule"—though its active head be a clergyman—I shall have very much overestimated the gravity of this Board's predicament. It may be admitted at the outset that this Board's powers were less than those of the Pennsylvania and Wisconsin Boards, although I shall show that its members did not exercise the powers given by the law which created it.

The Illinois State Board of Charities was created in 1869. The third section of the act creating it says: "The said commissioners shall have full power, at all times, to look into and examine the condition of the several institutions which they may be authorized by this act to visit, financially and otherwise; to inquire and examine into their methods of instruction, and the government and management of their inmates, the official conduct of trustees, directors, and other officers and employés of the same; the condition of the buildings, grounds, and other property connected therewith, and into all other matters pertaining to their usefulness and good management; and for these purposes they shall have free access to the grounds, buildings, and all books and papers relating to said institutions; and all persons now or hereafter connected with the same are hereby directed and required to give such information and afford such facilities for inspection as the said commissioners may require. § 4. The said commissioners, or some one of them, are hereby authorized and required, at least twice in each year, and as much oftener as they may deem necessary, to visit all the charitable and correctional institutions of the state, excepting prisons receiving state aid, and ascertain whether the moneys appropriated for their aid are or have been economically and judiciously expended; whether the objects of the several institutions are accomplished; whether the laws in relation to them are fully complied with; whether all parts of the state are equally benefited by said institutions, and the various other matters referred to in the third section of this act; and report in writing to the governor, by the fifteenth of December, annually, the result of their investigations, together with such information and recommendations as they may deem proper; and the said board of public charities, or one of them, shall make any special investigation into alleged abuses in any of said institutions, whenever the governor shall direct, and report the result of the same to the governor. § 5. The said commissioners, or one of them, shall also, at least once each year, visit and examine into the condition of each of the city and county alms or poor-houses, or other places where the insane may be confined, and shall possess all the powers relative thereto, as mentioned in the third section of this act." That the law contemplated more than a cursory examination is shown by the following sections: "The said commissioners, or any one of them, are hereby authorized to administer oaths and examine any person or persons in relation to any matters connected with the inquiries authorized by this act. Any actual outlay for any actual aid and assistance required in examinations and investigations, on being made out and verified by the affidavit of the commissioners making the charge, and approved by the governor, shall be paid by the treasurer, on the warrant of the auditor of public accounts, out of any moneys in the treasury not otherwise appropriated."

In the vicinity of the "Queen City of the West," as it has been exultingly termed, stands a large four-story brick building with diminutive windows, surrounded by numerous cracks and of a decidedly dilapidated appearance. This is the Cook County Hospital for the Insane. The insane in that state are still subjected in the county institutions * to treatment as vile as that of the days antecedent to Pinel and Chiarruggi. Of the early days of this institution there is but little history, but dark dungeons with chain attachments for the insane had been in use as late as 1875. This hospital's government is in the Board of County Commissioners, who name annually a Committee of Public Charities, who look after it and the county poor-house, of which, until a few years ago, it was a department, and is now on the same ground, though having a separate existence. The relations between the two are still very close. The Committee on Public Charities is composed of five members of the county board. The board elects the medical superintendent, the warden, the engineer and the druggist and assistant physicians; the engineer appoints his own assistants; all other employés are appointed by the warden. The influence of the county commissioners on these appointments is confessedly great. The Committee on Public Charities makes the purchases on requisition of the officers, usually after asking and obtaining authority to do so from the full board, though this is by no means an invariable rule of action; the bills are sent to the warden, who compares the items charged with the goods received and checks them as correct—with the prices paid he has no concern; the duplicate is retained at the institution, but the original must have the affidavit of the party furnishing the goods attached to it, and is submitted by the committee to the board, with a recommendation that it be paid, and it is paid after a vote approving the recommendation by

* CHICAGO MEDICAL JOURNAL AND EXAMINER, November, 1885.

the board, and placed on file in the county clerk's office. This organization varies in its details, at different times, according to the will of the county board or the committee on charities. From two-thirds to one-half of the county commissioners are low saloon keepers, and two of them supply with liquor two drinking-houses, licensed by the county board, in the immediate vicinity of the hospital and poor-house,—places of resort for the employés of both institutions.

An institution so ruled and governed, it is needless to say, should have been regarded with constant suspicion, and the results of any inspections brought constantly before the public; but the first knowledge the public had of the bad management of the institution was due to the self-sacrificing labors of Dr. J. S. Jewell, who in 1875 made a pains-taking investigation and found iron handcuffs in use, food of the vilest quality, bad drugs, bad clothing, drunken male and female officers and attendants, and dances of an improper character, and brought these facts before the Chicago Medical Society. The grand jury thereupon indicted the county commissioners, but from his efforts at reform, backed though they were by the feeling of the community, the State Board of Charities held stubbornly aloof.

A reform was made of a temporary character. Drs. Jewell, Brower, Lyman, and some medical politicians, were appointed an advisory board with the following powers: "It shall be the duty of the Board to confer with the Committee on Public Charities with reference to the condition and necessities of the asylum, and to make to the committee a quarterly report of the results of their investigation. They shall also, in conjunction with the Committee on Public Charities, recommend to the Board of Commissioners such persons for Medical Superintendent as, in their judgment, are suitable for such position. Any dispute which may arise concerning the medical management of the Institution shall be referred to the Advisory Board, in conjunction with the Committee on Public Charities, for investigation." But their recommendations were so persistently ignored that, worn out by circumlocution and insults, Drs. Jewell, Brower and Lyman resigned, and the ward political magnate once more reigned supreme over the insane of Cook county. Dr. Moyer, an interne during 1879–1880, states that the attendants were rough and brutal, the food poor and the medical supervision *nil.*

During 1883 the improper treatment of the patients and the character of the food were so notorious that more than one grand jury was anxious to investigate, but failed because they were repeatedly hoodwinked by the commissioners. Scurvy * was frequent and intoxication common. The death rate at this time was: males 23 per cent., females 24 per cent. The only American institution which that year had a death rate of more than 10 per cent. was the State Emigrant Insane Hospital, New York, which had a death rate of 17 per cent., the highest rate being among the females, and this was shown by legislative investigation to have been horribly mismanaged. According to the best authorities the average American death rate was 5–7 per cent.; and when it is much more than eight, in the absence of an epidemic, the increase is due to bad food, insufficient clothing and improper attendance.

At this time the State Board of Charities † was lauding the Superintendent for reducing restraint, although it was then used at the discretion of the attendant; although patients were

---

* Testimony of Mr. Harpel and Dr. Clevenger, CHICAGO MEDICAL JOURNAL AND EXAMINER, January to May, 1884.

† Report for 1883.

seated at the table for their meals in a straight-jacket; one jacketed, with a portion of food before her, eating it as a dog would. And the attendant, when spoken to about it, replied, "She always does so; even if she was not jacketed she would not eat any other way." But that very patient was taught to eat differently in 1885. Although working patients were treated like this: A very respectable mechanic had been employed to oversee the demolition of the old poor-house, had a room in the hospital, opportunity to observe the treatment of the patients there, as well as on the outside, when at work. It was a common thing for the attendants to threaten patients who would not work, with the straight-jacket. On one occasion a patient at work complained of being ill, and was continually going for water. The attendant said to him, "Come, Jack, if you won't work I will put the jacket on you." "I can't work." "Well, I will put the jacket on you." "Well, I'll take it, sooner than work." "Come with me." They went into the ward together, and Mr. H. followed after them, in a few minutes, when he saw Jack with the jacket on. The next day Jack came out again to work, but was unable to do so. The third day he stayed in the house; and the fourth day he was dead. The clinical clerk informed Mr. H., in answer to his inquiries, that he had died of typhoid fever; although attendants locked patients up for not doing fancy work.

They commended the Cook County Insane Hospital management as equal to the best State asylum; although no histories of cases by the physician in charge were kept; no census, and very meager records of any description; although the visits of the Superintendent to the wards were few and hasty; although in each ward was kept a bottle of whisky and a bottle of strong sleeping medicine of bromides and chloral, which the attendants dealt out at their discretion; although among attendants it was a common remark: "It is no use doing anything for these cranks;" although the physician was called only as a last resort, and informed of the condition of the patients and the attendant's therapeutics in the following language: "I gave her a drink of whisky and then a dose of sleeping medicine, but she did not get any better, so I called you;" although the real needs of the patients seemed to call for no thought; although they had no bath-towels, and attendants were in the habit of putting the clothing on the patients without drying the skin; although the wards were frequently cold, and the patients had no winter clothing. Many who would have been benefited by out-door exercise did not leave the ward for six months, because there were no wraps; although the quantity of flannel purchased annually did not average two yards for each patient;* although, to use the language of the Board † itself, the patients were so thinly clad in winter as to prevent any exercise in the open air, and to occasion great suffering inside the house, whenever, for any reason, the supply of heat was inadequate; although before the introduction of direct radiation (1884) it was impossible to heat the building properly; and, even after that improvement, the thermometer in some of the wards was at times as low as 55° F.; although the heating was unequal, sometimes insufficient and at other times excessive; although there were four rooms in the west wing into which it was impossible to force hot air; although straw beds were the rule, hair mattresses the exception; although great difficulty was experienced in obtaining straw, and hay had sometimes been used instead; under these circumstances, the straw cannot have

---

* Testimony of Mrs. Lowell, Dr. Howe, Mr. Hughes, Mrs. Welker.

† Report of the Board for 1886.

been renewed as often as desirable; although the number of blankets was but four pairs to every three patients; considering the destructive habits of the insane, and the want of sufficient stock on hand, this allowance should have been at least one pair a year to each patient; although there were but few quilts; although the clothing and cleanliness of the patients received but little attention, and for weeks and weeks the patients were without fine-tooth combs; although diarrhœa and scurvy* were frequent and but little attention was paid to the diet; sick patients were fed with the same food as the others. The great article of diet was pigs' heads, boiled without being shaved or cleaned. Hogs' ears, with the hair on them, were often set before the patients to eat. Bunches of hair half as large as a little finger were to be picked out from the patients' food. Although dying patients were fed on sour milk; although the milk, which is so great a necessity to the treatment of the insane, was almost never fit for use; although they had meat never more than once a day, and often not that, and the meat frequently stank; although incidents like this were frequent: Mr. H. observed it one day, when its appearance led him to ask the butcher, "What do you do with that?" "That's for the cranks." "In the name of goodness, you don't cook that for them?" "Oh, that's good," he replied, "they don't know the difference;" although patients died from innutrition, owing partly to their own indisposition to take food and the neglect of attendants to feed them; although the quality of the drugs may be judged from the fact that the mercurial ointment contained but ten per cent of mercury; although Saturday night dances already mentioned were marked by the conduct described

* CHICAGO MEDICAL JOURNAL, January, 1884.

years ago by Dr. Jewell. Every Saturday and Wednesday a dance was held, and beer kegs were frequently brought up into the dance hall and emptied by the attendants and employés after the patients had retired, festivities being kept up till morning.*

Although the superintendent† in charge avowed as a settled principle that he did not believe in watching his employés, and this at a time when appointments in the institution were made more on account of politics than on account of qualifications for the positions, and the appointments were chiefly made by the Chicago Boss, a prominent gambler. The Board itself says: "Insane people are, from the nature of their malady, unreasonable, exacting and irritating, sometimes dangerous; attendants often feel a contempt for them, especially if they belong to the pauper class; and, in an institution like Dunning, there will always be unkindness on the part of certain attendants, which will be heightened if the discipline is lax. A large part of the duty of every physician of an insane hospital consists in watching attendants, weeding out the incompetent and unfaithful among them, and supplying their places."

With such an avowed policy by a superintendent as the above, with the fact of an enormous death-rate, with the character of the persons making appointments, with the previous history of the insane-hospital, the notoriety of the abuses in mind, it must be obvious that a careful, exact, suspicious investigation every year was absolutely necessary; but the examination on which all these laudations were based

---

* Evidence of Dr. Howe, Dr. Clevenger, Mrs. Lowell, Mr. Hughes, Mrs. Welker, Mrs. Heintz.

† Testimony of Dr. Spray.

was made, not by a commissioner or the secretary, but by the accountant of the Board.

September, 1884, Dr. J. G. Kiernan was elected medical superintendent, and a warden, an ex-gambler, was elected, who had charge of the financial and domestic management. On Dr. Kiernan's entrance he found that more than one-half the employés were appointed on the demand of a county commissioner, the keeper of a low gin-mill, the lieutenant of the boss of Cook county, the gambler before-mentioned. One-half of these employés had been there for years.* The male attendants were, as a rule, coarse, brutal men, and within two months Dr. Kiernan discharged five of them for striking patients; receiving his first taste of the discipline hitherto prevalent in the institution by being knocked down and called a "crank" for interfering with one individual amusing himself by pounding a dement. . . . . The engineers and other mechanics had keys to the female wards, and frequently visited them in an intoxicated condition, often cursing the attendants. There was no means of determining just what patients were in the building, and, as a careful census revealed, there were four more patients on the register than in the institution. In a remote ward patients were found with ulcers swarming with maggots, and none of the patients of the ward had been out for months. The drug store was destitute of the most necessary articles, there being but one drachm of quinine in the whole building, and many patients ill from low types of intermittent fever. The drug store was a gin mill to which employés invited the politicians who visited them. The druggist, a

* Testimony of Drs. Spray, Kiernan, Howe, Clevenger, Mrs. Lowell, Mrs. Heintz, Mrs. Welker, Mr. Hughes.

conscientious, able pharmacist, complained bitterly of being turned into a bar-tender, as he did also of the vile drugs sent him.

Dr. Kiernan asked for 300 overcoats and 300 shawls; the committee allowed him of each fifty. He wanted fine combs, and could not get them, nor could he procure fresh vegetables. The peculiar nature of the institution may be judged from the following facts: Poker-playing was carried on in a room in the basement, where cigars are sold. Both male and female employés sat on the stairs in dark passages at night. Male employés were in the habit of visiting female attendants socially, in their wards, in the evening after the patients had retired. Keys to these wards were given to the mechanics employed about the building, and the character of its discipline may be judged from the following incidents: On one occasion there was a row between a male attendant and the laundryman; on another, the assistant engineer slapped a female attendant in the face. Dr. Kiernan said that male employés should not go through the female wards; Mr. Kavanaugh, the engineer, said that they should. Dr. Kiernan was knocked down by an attendant, struck by the engineer, and choked by the night watchman, so that he had a hæmorrhage and was confined for some time to his bed. Some unknown person fired a shot into Dr. Clevenger's room, on the night after the presidential election, and the ball was stopped by a book in the book-case a few feet from his wife. Disobedience to orders was not always followed by discharge. Dr. Kiernan had power, for three months after his appointment, to discharge attendants; then it was taken from him. The housekeeper, Miss McAndrews, took a patient suffering from some female disorder out of her ward and set her to scrub, in violation of orders; when Dr. Kiernan expostulated with her, she replied, "I do not propose to have anything to do with you or your orders." The whole medical staff united in a request for her discharge, but it was refused; Commissioner Leyden said that if Dr. Kiernan continued to insist upon her discharge he would make it hot for him, which he proceeded to do. In consequence of the horrible cooking, Dr. Kiernan tried to procure the discharge of the cook, but failed. This cook threatened "to lay the doctor out." Miss Finerty had been cautioned that a certain patient was not to be left alone in the ward, but taken out when the rest were; she disobeyed, left her in her room, and while there she hung herself; in consequence of this suicide there was an investigation by the Committee on Charities, when the warden and Commissioner Van Pelt said that she should be discharged, but Commissioner J. J. McCarthy said that she should not be, and she was not, and is in the institution now. A Miss Thornton, a sister of the boss gambler's bar-keeper, in charge of an epileptic ward, was found by Dr. Kiernan repeatedly in bed asleep, and the ward-keys in the hands of the patients, but despite his repeated requests she is still an attendant. It is not surprising, therefore, that such employés testified that he was very unpopular. They thought that he required too much of them, and that he was too ready to ask for their dischage.* One special ground of complaint was that he did not allow the attendants every other afternoon off duty, when in no institution in the world are attendants allowed every afternoon off duty, and the number of attendants, not excluding those off on leave, in the Cook County Hospital for the Insane is but one to sixteen—much

* Testimony of Miss Thornton.

too few in any case. Commissioner Ochs testified that Dr. Kiernan was unpopular because the employés thought he was too strict about restraint and the treatment of patients.

Persons so guarded by political influence would naturally look upon any inspection of them as an insult, and it is not very surprising that when a supervisoress * attempted to perform the duties incumbent on such officers, the result should be that in a few months the warden* called her into the parlor, to speak to her in private, when he said: "I would rather part with my right arm than tell you what I have got to say to you. I shall have to ask you for your resignation. You have fulfilled your duty here in every respect. There is no cause for your dismissal, but you are not popular with the attendants—the simple fact is, you are too much of a lady to be here. It is either you or I: you will have to go, or I will have to go. You are not popular with the commissioners; they do not know you, and the plain truth of the business is, you are not in the ring." Of course her successor took very good care to be popular with the attendants, whose general character was *well* summed up in the fact that while there were some who were most excellent, conscientious, and endeavored to mitigate the sufferings of the insane in every way possible, they were in a minority, and overawed by those who were not inclined to do their duty.† The character of the female attendants, as a rule, may be judged from the fact that to the two drinking places already mentioned a female attendant took two female patients, at different times, and treated them there. In the fall of 1884 the condition of the asylum was a matter of public discussion; since in October, 1884, Dr. S. V. Clevenger, then pathologist of the Cook County Hospital for

* Testimony of Mrs. Phillips.

† Special Report of State Board of Charities, 1886.

the Insane, read a paper before the Chicago Medical Society, in which was embodied most of the facts already cited from the report of the Board of Charities. In consequence, the Chicago Medical Society and the Citizens' Association united in requesting the Illinois State Board of Charities to unite with them in investigating; but no action was taken, although the Chicago daily papers contained published interviews in which Drs. Howe*, Koller* and Kiernan,* of the Asylum, demonstrated the truth of Dr. Clevenger's statements, as well as did published recommendations of Dr. Kiernan to the Local Charities Committee. Scurvy was then present to a limited extent, but as Dr. Kiernan was unable to procure fresh vegetables a serious epidemic of scurvy occurred about February, involving about two hundred patients, all of whom were permanently injured by it, and several died from its indirect influence; in two cases death resulted from it. The character of the food during Dr. Kiernan's time is thus summed up by the State Board of Charities: County Commissioner Klehm considered that the fare was too meagre, that the supper especially was not sufficient, that the only full meal given was dinner, and that there was a lack of vegetables, to which he drew the attention of the warden and of the chairman of the committee. Dr. Alexander said that persons in health might support life on the food given, but it was poorly cooked, and, for insane people, unwholesome and insufficient in nutritive value. Dr. Kiernan had the same medical opinion of it, and made persistent but ineffectual efforts to have a proper diet list established. Commissioner Wasserman moved, in committee, December 12, 1884, that the list proposed by Dr. Kiernan be adopted, and the warden in-

* See particularly Chicago *Daily News*, Nov. 16, 1884; Chicago *Daily Tribune*, Nov. 29, 1884.

structed to see that it be conformed to by the cook; the motion carried, but nothing came of it. As to the quality of food, the bread was good; the coffee not to be distinguished from the tea. The cooking was bad, and the facilities for cooking, for the number of patients fed, were wholly insufficient; although thousands of dollars were being spent on a fence and a greenhouse, and repairing the officers' dining room. It is not possible to serve anything but a boiled dinner, which, in the quaint language of the assistant cook, is not properly cooked, but "coddled." The service was bad; there was a failure to distribute the food properly, so that some wards received too much and others not enough; the table furniture generally uninviting; in many wards tin plates and cups are used, and the ladies of the Women's Club had repeatedly seen them black and dirty; the conduct of the patients at their meals was disorderly; the attendants ate in the officers' dining-room in the center building. Pigs' heads were a frequent article of diet. Meat is furnished, under a contract, by a butcher (Bipper) whose bills for 1884, were $15,763.04, and for 1885 they were $18,934.11. They contain hogs' heads, charged and paid for at nine cents a pound, the price of good pork. The assistant cook had never seen joints of pork brought in for the patients, and when asked in what condition the heads were when brought to the kitchen, replied, emphatically, "brought into the kitchen a disgrace to the butcher who sent them." Asked to explain his meaning more fully, he said that the hair had not been properly scraped, when scalded, and that it was embedded in the skin so that it could not be removed by the knife nor by fire. Dr. Alexander said that they never were inviting—the bristles were very long. Dr. Howe had seen black dirt on the ears, which the cooks had not succeeded in boiling off. In June, 1885, a patient found in her plate an iron-ringed, uncleaned catarrhal snout, which led to an investigation by the committee, and the snout and ring were exhibited to Commissioners Lynn and Van Pelt, in the warden's parlor Commissioner Van Pelt directed the discharge of the cook, but his order was disregarded.

As before, the State Board of Charities held aloof and did not even visit the institution, as the law requires, that year. Sept. 1, 1885, Dr. Kiernan's term expired. October 19, 1885, the condition of the Cook County Insane Hospital was once more brought before the Chicago public. Although the State Board of Charities was then in session in the city, no action was taken by it until upon the request of the leading journals, of the Chicago Woman's Club, of members of the Bar, of the Citizens' Association, and of the Chicago Medical Society, the Governor ordered the State Board of Charities to make an investigation. The result of that investigation was such that a strongly condemnatory report of the condition of the asylum was made. Although every charge made in the paper of Dr. Clevenger, read in 1884, was sustained, the Board claims that: "The results arrived at by us were mainly the results of the double-headed management in Dr. Kiernan's time; and it was an extraordinary sight to witness his willingness, at the risk of censure which ordinarily might be visited upon him personally, to reveal the defects and failures of his own administration. We give him credit for both courage and public spirit in the course pursued by him."

The question naturally arises, Is the cause assigned by the Board sufficient to account for the evils which have existed for the past two years in the Cook County Insane Hospital?

The Board says: "It was shown that political considerations very largely governed the election of officers by the Board, and the appointment of employés by the officers; that instead of intrusting the government to the Superintendent, the Commissioners themselves spend an unnecessary amount of time at the hospital; that conflicts arise between the officers as to the limit of their respective powers; that there is much insubordination on the part of the employés; that the discipline is lax; that supplies, when needed, cannot be easily and speedily procured; that the patients suffer various inconveniences in consequence, and that it is often difficult, if not impossible, to estimate the responsibility of individuals for the evils which are seen and admitted to exist." But it admits that all these evils existed before the dual management was instituted, and a careful examination of the testimony cited in its report will show that nearly all the evils proven existed long before the dual management. If Dr. Kiernan was not afraid of censure in exposing the defects of his own administration, it is obvious that his evidence becomes of double value as to the condition of things he found in the institution, already cited from his evidence. The testimony of Mr. Hughes as to the treatment of typhoid fever cases by enforced labor by means of the camisole related wholly to the period before the dual management, as Mr. Hughes' connection with the institution ceased July, 1884, and the dual management began Sept. 1, 1884. The evidence of Mrs. Lowell related almost wholly to that period, and that of Mrs. Welker, Mrs. Heintz, Dr. and Mrs. Clevenger, Dr. Howe and Mrs. Shedd largely to that period. The Board itself uses the following language : "Under Dr. Spray the system of administering medicine was objectionable. Whisky and sedative mixtures were sent up in large quantities marked "ward use," and considerable latitude allowed to the attendants in their administration. They then cite the evidence of Dr. Delia E. Howe, who says, after stating the therapeutic measures already mentioned as used by attendants: "It took some time to impress the idea that I preferred to be called before the ever-ready remedies were used. Evidently a physician had been a luxury and only called as a last resort. Attendants hired patients to work for them by giving whisky and sleeping medicine, which these patients had come to crave as opium-eaters opium. The amount of sleeping medicine and whisky used in the female wards alone was enormous. That both male and female attendants helped themselve quite largely from the ward whisky-bottle, which was filled whenever they desired, is beyond doubt." These practices were stopped by Dr. Kiernan, but resumed after September 1, 1885.

Dr. Koller once saw an attendant (who had been in the asylum six years) strike a patient in the face, because he would not go to dinner. A former patient (during 1883–84) saw a female attendant (six years in the asylum) kneel on a patient's stomach and try to pour a dose of medicine down her throat; at another time she saw an attendant (six years in the asylum) slap a patient, who had on a straight-jacket, in the face, because she persisted in calling for Dr. Spray; she also saw an attendant pull a patient out of the dining-room by the hair, slap her and strap her to a bench, for breaking a plate. The housekeeper, who compelled very sick women to do scrubbing, despite the Doctor's orders, had been in that position two years, and had been an attendant for three years before. It was in evidence that the superintendent, during 1883, himself had on more than one occasion struck female patients. It was in evidence that drunkenness was common in the asylum during 1883–4; that some of the commissioners frequently visited it in an intoxicated state. The drug store at all times was a gin mill. The Board says: "The liquors at Dunning are kept in the drug-room, and dispensed with great freedom to visitors, officers, and even to employés. Intemperance on the part of the employés, in numerous instances, was clearly proved, among the rest, a male night-watch, an assistant engineer, and several attendants. Miss Lowell, 1883–4, had seen two female attendants intoxicated. Dr. Howe went into the drug-room one night, and found female attendants drinking beer there." And this during 1883–4. The Board of Charities itself states that the same condition of insufficient clothing existed in 1883–4 as in 1884–5. That scurvy existed during 1883–4, and everything already stated as to that period will be found in the report of the board for 1886. The Board says, concerning the system of government, that: "The county board, it is true, after electing as many officers as it may think expedient, turns the management of the asylum over to a committee; but the relations of this committee are very different from those of a board of trustees, since the members of the committee which has to account for the management are also members of the board to which the committee must account. As commissioners, they necessarily form a part of every combination, political or otherwise, in the larger body to which they belong, and their action as trustees or committeemen is controlled by the exigences which grow out of such combinations, rather than by a disinterested regard for the welfare of the inmates of the institution of which they have charge. What powers they do possess should, under the law, be exercised by the committee as a unit, and their acts should be made a matter of record; but they have no secretary, keep no records, and each member of the committee assumes, or may assume, to exercise, individually, the power which in fact is vested only in the full committee, regularly convened. The county board is a changing body; it has no opportunity to inform itself as to the nature of insanity, the needs of the insane, the principles of organization, or the practical methods of work best adapted to the successful management of a public institution; experience has demonstrated that it does not even know what a public institution ought to cost for maintenance, nor how the items of expenditure should be apportioned. The county board is, of course, a political body; it is elected by popular vote, and it is only natural to conclude that the positions within its gift will be bestowed in part with a view to the political effect of such appointments upon the personal fortunes of the commissioners. How can it be otherwise? It is equally natural to suppose that those who have successfully labored to secure an election by the board will be more or less subservient to the wishes of the commissioners in making subordinate appointments." Yet this system was present during 1883–4.

It is well known in Chicago that in 1884 a political quarrel occurred in the dominant party; some few set up as reformers, and it was through these and certain actual reformers Dr. Kiernan was elected superintendent on medical recommendations, he being personally unknown to them. The real reforming element was at the time desirous of changing the management of the asylum. Two men, much respected for their personal integrity, Commissioners Senne and Klehm, although holding different political views from Dr. Kiernan, strongly supported him, then and thereafter. The attempts at reform of Dr. Kiernan soon drove the sham reformers and ring men into alliance, and the number of the anti-ring men was diminished by three, on account of an election during Dr. Kiernan's term,—a lawyer of prominence being succeeded by a commissioner best known by his frequent arrests for brawling in saloons; whose character may be judged from what is already cited from the State Board of Charities' report concerning him. The Board, during 1883, was dominated by the gambler "Boss" already mentioned.

With the facts already mentioned in mind, it is obvious that the State Board of Charities was desirous to screen the administration during 1883-4. It could not have been for political reasons, for during 1883-5, the same *party* was theoretically dominant. It could not have been from a bias in Dr. Spray's favor alone, for the Board itself condemns his medical system, while it commends Dr. Kiernan for changing it. The reason is evident from its course during the investigation, which it limited to a period subsequent to the examination made by its accountant, in the summer of 1883, when it enforced the legal rules of evidence against Dr. Kiernan, who brought charges which it now admits were true, but failed to enforce those rules in his favor when it permitted the asylum managers to introduce rebuttal evidence in the midst of his direct evidence, and to then make mendacious attacks on his character, but refused to allow him to introduce any direct evidence after the rebuttal evidence of the accused, although it was well aware that it had rendered him no assistance in procuring witnesses,—a matter of difficulty without its aid. It refused at first to allow him legal aid, although the county attorney acted as counsel for the defense, and treated himself and his witnesses at first with severity. Reference to the law will show that the Board did not make any inspection of a legal character in 1883. That it failed to make even a pretense of an inspection in 1884 and

1885 is obvious from its own report. The facts discovered in 1885 might have been revealed in 1883, and much suffering saved the hapless inmates of the Cook County Hospital for the Insaneduring the two following years. With the request for an investigation made by Mr. Ambler, secretary of the joint Chicago Medical Society and Chicago Citizens' Association Committee on the Insane Asylum Abuses in 1884, in mind, it is difficult to understand why the Board, if desirous of doing its duty, should fail to comply with the law in that and the following year. "From an impartial examination of the above evidence, including the report of the Board named, I believe the following conclusions naturally follow: 1st. That at a time when the Cook County Asylum was regarded as a place where "ward workers" and certain female acquaintances of ward politicians were provided for under the pretext of serving as attendants, and its higher offices were filled by drunkards, gamblers and ex-concert saloon-keepers; when the unfortunate inmates were delivered over bound hand and foot, as it were, to the mercy of the most vile, filthy and brutal of their species; when for every dollar stolen *per diem* there was an insane patient shivering or dying for want of clothing; and when for every commissioner and employé who became intoxicated on asylum "supplies" there was a case of scurvy in the wards, the Illinois State Board of Charities commended the management in eulogistic terms, basing these laudations on an illegal inspection. 2nd. That when public indignation was aroused, when it became evident that the grand jury were periodically hoodwinked, when almost every issue of every newspaper throughout the land was filled with revolting details illustrating the mismanagement of the Cook County Asylum, and the Chicago Medical Society and Citizens' Association united in requesting the Illinois State Board of Charities to inspect matters, it did not even order a formal legal inspection. 3rd. That when a physician whose name is well known to European and American students of insanity, having been urged to attempt a reform of the Cook County Asylum supported by the best medical, legal and social elements of Chicago, was met at every step by organized opposition, and on one of several similar occasions almost fatally injured for venturing to interpose between a helpless lunatic and a brutal attendant crazed by drink, he received neither support nor encouragement from the Illinois State Board of Charities. 4th. That it was not until the Citizens' Association, Women's Club, a Chicago Bar Committee, the Medical Society, the editors of influential journals and leading citizens induced the Governor to order the State Board of Charities to do its duty, that it reluctantly proceeded to its performance. 5th. That the Illinois State Board of Charities in conducting the examination displayed a hostile feeling toward those who had pressed the examination, hampered the giving of evidence as to abuses, while favoring, indirectly, malicious and mendacious attacks upon private character of witnesses to such abuses. 6th. That the report confirming the existence of gross abuses which the State Board of Charities found itself compelled to make as a result of the investigation it had so reluctantly begun, convicts the above Board of having failed to perform its duties since its organization, and of being, therefore, directly and indirectly responsible for untold misery endured by the helpless thousands who had no other body to look to for relief, but who, during these years of mismanagement, plunder, cruelty and neglect, looked to this body in vain. 7th. That inasmuch as the Illinois State Board of Charities' report confesses to a condition of affairs from 1883 to 1886 which under the circumstances (as is proven by Dr. J. S. Jewell's earlier report) could have been but the consistent continuation of a similar state of affairs prior to 1883, this proves that its eulogistic report of that year must have been false in fact. 8th. That in view of this, the arbitrary limitation of the investigation by the Illinois State Board of Charities to the period subsequent to the report of 1883 was a manœuvre as to whose objects there cannot be a doubt in the mind of any reasoning person. 9th. That the State Board of Charities does not ensure the protection of its unfortunate wards, but, on the contrary, through its opposition to reform, when proceeding from sources not under its immediate control, its ignoring of well founded and well supported requests for investigation and remedial action, its encouragement of personal squabbles to the detriment of vital evidence, and its misleading reports, commending what it was instituted to expose and condemn, constitutes the best protection the asylum politician can desire, is to be regarded as the guardian of incompetency in the medical, of fraud in the financial and of brutality in the administrative management of insane and other asylums. 10th. That for the benefit of the insane of Illinois, a body more inclined to carry out the provisions of the act of 1869 is needed. 11th. That in all large states three State Commissioners in Lunacy, two of whom should be alienists and one a lawyer, should be given the duty of supervising the insane."

